# Assessment of the mechanical forces applied during eye rubbing

**DOI:** 10.1186/s12886-020-01551-5

**Published:** 2020-07-22

**Authors:** Farhad Hafezi, Nikki L. Hafezi, Bojan Pajic, Francesca Gilardoni, J. Bradley Randleman, Jose Alvaro P. Gomes, Léonard Kollros, Mark Hillen, Emilio A. Torres-Netto

**Affiliations:** 1 Laboratory for Ocular Cell Biology, Center for Applied Biotechnology and Molecular Medicine, University of Zurich, Zurich, Switzerland; 2 ELZA Institute, Dietikon, Switzerland; 3 USC Roski Eye Institute, University of Southern California, Los Angeles, CA USA; 4 Faculty of Medicine, University of Geneva, Geneva, Switzerland; 5 Department of Ophthalmology, University of Wenzhou, Wenzhou, China; 6 ORASIS Eye Clinic, Swiss Eye Research Foundation, Reinach, Switzerland; 7 Department of Physics, Faculty of Sciences, University of Novi Sad, Novi Sad, Serbia; 8 Faculty of Medicine of the Military Medical academy, University of Defense, Belgrade, Serbia; 9 The Cleveland Clinic, Clevelan, OH USA; 10 Department of Ophthalmology and Visual Sciences, Paulista School of Medicine, Federal University of Sao Paulo, Sao Paulo, Brazil

**Keywords:** Corneal biomechanics, Keratoconus, Eye rubbing

## Abstract

**Background:**

To determine the average amount of mechanical forces applied to the lids of keratoconus patients during eye rubbing.

**Methods:**

Fifty-seven patients (41 male, 16 female, average age 34.8 years) with a clinically and topographically diagnosed keratoconus and a history of eye rubbing were prospectively asked to perform their individual eye rubbing movement on a high-precision balance. The type of eye-rubbing movement and the force applied, represented in newtons (N), were recorded and analyzed.

**Results:**

We detected three different types of eye rubbing. Rubbing with the fingertip was most frequent (51%), followed by rubbing with the knuckle (44%) and rubbing with the fingernail (6%). Each type of eye rubbing showed different average forces, with knuckle type eye rubbing applying significantly more force (9.6 ± 6.3 N) on the lids than fingertip (4.3 ± 3.1 N) and fingernail (2.6 ± 3.3 N) types (*p* < 0,001 and *p* = 0,016, respectively).

**Conclusions:**

There were major variations in the force exerted on the lids, depending on the type of eye rubbing employed. This data will help determine the forces that need to be applied in future experimental eye rubbing models.

## Background

Keratoconus (KC) is a potentially sight threatening corneal disease characterized by a progressive corneal protrusion with irregular astigmatism and corneal thinning. Primarily a disease of the collagen of the cornea, the classification of keratoconus as a rare disease, as suggested by Kennedy et al. in 1986, is being reconsidered in the light of increasing prevalence when using more modern measurement techniques such as topography and tomography [1, 2].Keratoconus represents one of the most frequent causes of severe visual impairment in children and adolescents. The exact etiology of keratoconus remains unclear [3]. Besides genetic predisposition, intense and prolonged eye rubbing may play an important role in the pathogenesis of the disease. Repetitive and prolonged eye rubbing may alter corneal biomechanics and trigger keratoconus onset or exacerbate its clinical presentation, as postulated in a number of recent publications [4–6].

Patients who intensely rub their eyes (e.g. those with Down Syndrome and atopic patients are at higher risk of developing keratoconus [4, 7–9]. The reasons to rub their eyes are manifold: itchiness in atopic conjunctivitis represents one of the most common causes of eye rubbing [10]. Also, “removal relief” eye rubbing is observed frequently in contact lens wearers [5]. Lastly, behavioral eye rubbing can be observed in a wide variety of forms, ranging from occasional eye rubbing in healthy subjects to more severe forms of repetitive eye rubbing in patients with developmental disorders (oculo-digital sign) or psychiatric affections (obsessive-compulsive eye rubbing) [11, 12].

Patients rub their eyes in a number of different ways, i.e. by using the pulp of their fingertips or their fingernails. Others use their knuckles (proximal interphalangeal joint). Also, the strength and the intensity of eye rubbing may vary [5]. We performed this study in patients with keratoconus to assess average forces typically applied to the globe during eye rubbing.

## Methods

This prospective study was conducted on out-patients seen for a clinical follow-up of keratoconus at the ELZA Institute in Dietikon/Zurich, Switzerland, between June 2018 and June 2019. The Institutional Review Board of the Canton of Zurich approved the study protocol, which adhered to the tenets of the Declaration of Helsinki, and written informed consent was obtained by all participants before inclusion.

Fifty-seven patients were included, 41 were men and 16 were women. The average age was 34.8 years (range 17 to 42 years). The inclusion criteria were the topographic diagnosis of keratoconus and a history of eye rubbing. All patients with pre-existing ocular disease other than KC or history of ocular surgery were excluded.

All subjects had bilateral corneal assessment performed using a rotational Scheimpflug system (Pentacam HR, Oculus, Wetzlar, Germany). Since there is ongoing discussion about the best approach for keratoconus classification and to avoid inclusion of borderline cases, KC diagnosis was based on a stringent profile (focal steepening of more than 3D, coincident focal paracentral thinning, and a BAD-D index of more than 2.0) used previously [2].

**Fig. 1:**
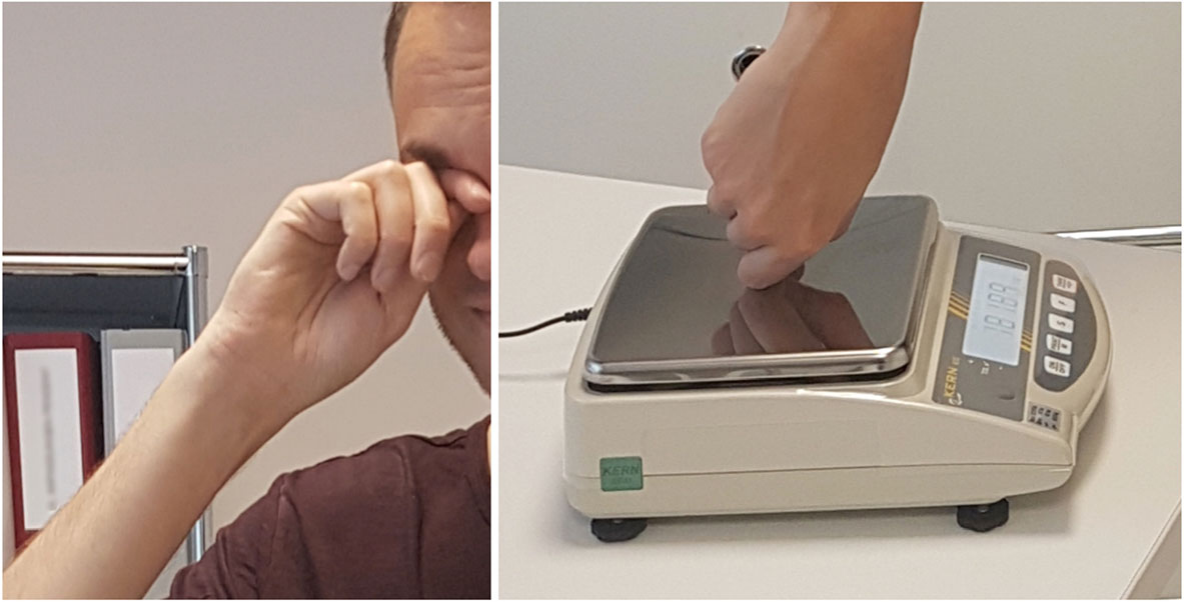
Patient performing “knuckle-type” rubbing on his eyelid (left) and on a high-precision scale (right)

Patients were asked to perform their individual eye rubbing movement and then repeat the same movement on a surface of a high-precision balance (Kern EW, Kern & Sohn GmbH, Balingen, Germany) (Figure 1). Applied force measurements represented in newtons (N) were taken in quintuple. To assess repeatability, patients were asked to step away from the scale after each eye rubbing recording and reposition themselves for every measurement. For every measurement, patients rubbed for 10 s and the highest recording was maintained. The mean of the five measurements was used for analysis. Similarly, we noted which part of the finger came into contact with the eyelids every time an eye-rubbing movement was performed. Hand dominance was recorded by asking the patients which hand was used for writing.

Statistical analysis was performed using IBM’s SPSS Statistics v.24 (IBM Corporation, Zurich, Switzerland) and Microsoft’s Excel program (Microsoft Excel version 16.23 for Mac). Normal distribution was tested with both the Shapiro–Wilk and Kolmogorov-Smirnov tests. Non-parametric Mann-Whitney test was used to verify statistical significance. A confidence interval of 95% was used to determine significant differences between groups. Differences were considered significant whenever the *p*-value was less than or equal to 0.05.

## Results

Data were prospectively collected in a total of 57 keratoconus patients with a history of eye rubbing. Forty-one were male, and 16 patients were female. The mean age of the patients was 34.8 years, ranging from 17 to 42 years.

All patients included in the present study had keratoconus in at least on eye with focal steepening of more than 3D, coincident focal paracentral thinning, and a BAD-D index of more than 2.0. Although the objective of the current study was not to evaluate tomographic data, Scheimpflug data showed that flattest, steepest, mean and maximal keratometry, on average, were respectively 45.6 ± 5.6 D, 48.2 ± 6.3 D, 46.9 ± 5.9 D and 52.6 ± 8.4 D. On average, thinnest corneal pachymetry was 449 ± 65 μm, Q-value asphericity was − 0.64 ± 0.45 and BAD-D value was 7.9 ± 6.3.

Fifty patients were right-handed, and 5 patients were left-handed. In 52 cases, patients used either their dominant hand (*n* = 24) or both hands (*N* = 28) to rub their eyes. In the 3 remaining cases, patients were right-handed, but used their left hand to rub their eyes.

**Fig. 2:**
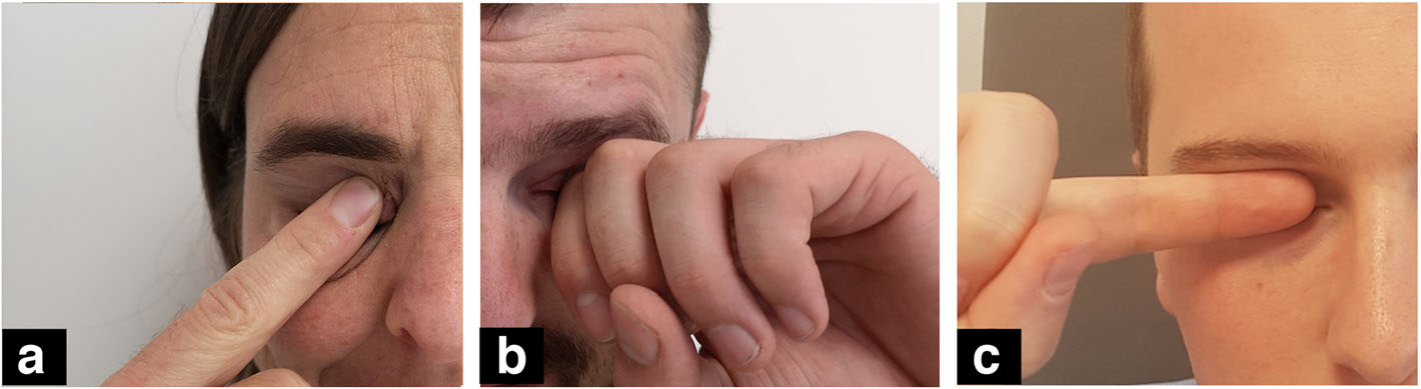
Types of eye rubbing. **a**, finger pulp; **b**, knuckle; **c**, fingernail

Three different types of eye rubbing were identified: “fingertip” rubbing, in which the patients used the pulp of their finger to rub the eyes, “fingernail” rubbing and “knuckle” rubbing, both using the proximal interphalangeal joint (Fig. 2). While finger-pulp rubbing was the most prevalent type of movement found in our sample, few patients used their fingernails. The number and ratio of each movement as well as the forces generated by the three types of eye-rubbing are detailed in Table 1.

**Table 1:**
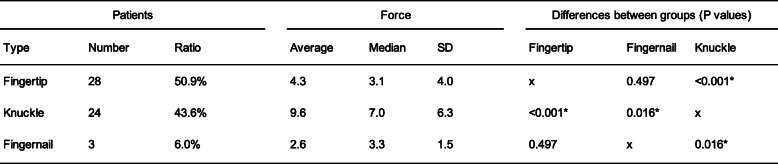
Forces applied on the closed eyelids in finger pulp, fingernail and knuckle-type eye rubbing

**Fig. 3:**
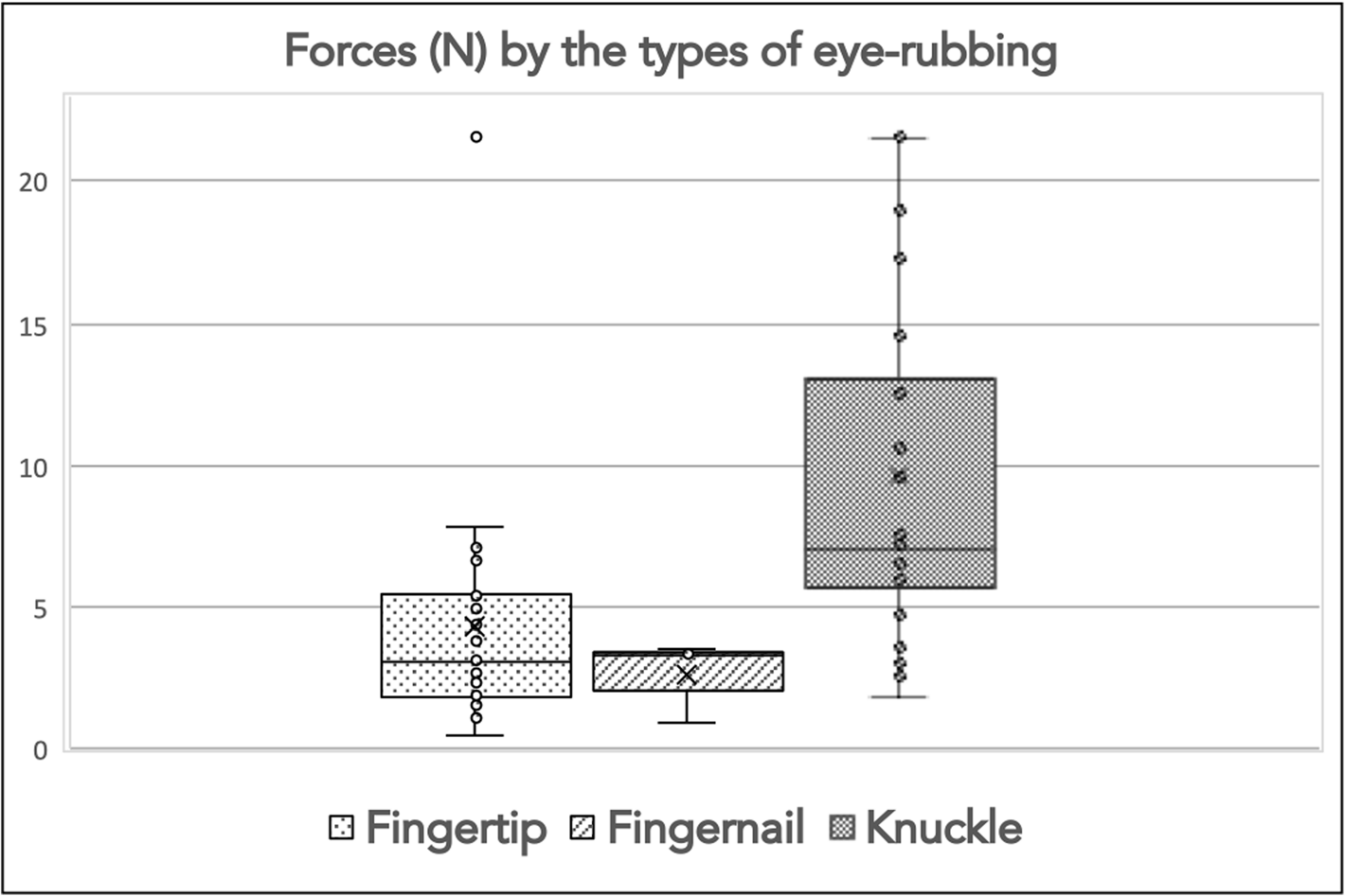
Forces (N) exerted by the different types of eye rubbing

The greatest force was exerted when the knuckle was used, in average 2.2 and 3.7 times greater when compared to fingertip or fingernail eye-rubbing, respectively. These differences were significant (Table 1). The individual variability for each type of eye rubbing is displayed in the Whisker-chart (Fig. 3).

## Discussion

Eye rubbing is a well-known risk factor for the progression of keratoconus, but little is known about the impact of repetitive eye rubbing on corneal biomechanics. In this study we asked keratoconus patients to reproduce the eye rubbing movement and strength on a balance.

The scope of this study was not to investigate a potential correlation between the severity or location of keratoconus and the type, the strength, the frequency and duration of eye rubbing, nor with the handedness of the patients. The latter was rather recorded to assess whether the patient used the dominant or the non-dominant hand for the eye rubbing measurements.

Rather, the aim of our study was to gather information on the extent of physical forces exerted on the globe during eye rubbing in keratoconus. The data gathered here are pertinent to our ongoing research and the physical forces recorded will be used in an experimental eye rubbing model, investigating the effect of repeated eye rubbing on corneal biomechanics (manuscript in preparation).

We detected major variations in the force exerted on the globe during eye rubbing. Of all types of eye rubbing, knuckle rubbing exerts the highest force on the globe. In this study’s group of patients with keratoconus, those who rubbed their fingernail exerted the lowest force, whereas patients that rubbed their eyes with the fingertips applied an intermediate force. Thus, the force exerted on the eye in rubbing is strongly related to the eye rubbing type.

Although only the mean of the 5 measures was used for analysis, variability between the 5 measures of the same patient was between 5 to 10% only. On the other hand, among the types of eye-rubbing, a wide range of values could be observed and are represented in Fig. 1. One point to be emphasized is that of the 57 patients, only 3 used their fingernail to rub their eyes. While this illustrates that such type of movement is not frequent in our patient series, this small number may be a limitation for comparison purposes with other groups.

One possible limitation of the study refers to the measurement method. Ideally a measure of pressure on the ocular surface should be taken whenever the patient performs an eye rubbing movement. Since no commercially available device is available for this purpose, a high-precision scale was used. Although ergonomics does not completely represent a physiological condition, it was attempted to reduce this bias by asking the patient to first make the movement on their eye and to immediately repeat this movement on the high-precision scale. Another limitation is that eye rubbing force is a vector function of time and position, also involving shear force. The amount of shear force cannot be measured by the simplified model used here.

## Conclusions

In conclusion, there were major variations in the force exerted on the lids, depending on the type of eye rubbing employed, with knuckle-based rubbing showing the greatest forces. In the future, experimental eye rubbing models can be used in order to better understand the impact of repetitive eye rubbing on corneal biomechanics and the potential correlation between applied force and keratoconus levels. Our data gathered here will help determine the amount of force that should be used in such models to mimic the human condition.

## Data Availability

All data generated and analyzed during this study are included in this published article. The datasets used and/or analyzed during the current study are available from the corresponding author on reasonable request.

## Abbreviations

BAD: Belin/Ambrosio Enhanced Ectasia Display
KC: Keratoconus
N: Newton
